# 3D Printed Sand Tools for Thermoforming Applications of Carbon Fiber Reinforced Composites—A Perspective

**DOI:** 10.3390/ma14164639

**Published:** 2021-08-18

**Authors:** Daniel Günther, Patricia Erhard, Simon Schwab, Iman Taha

**Affiliations:** 1 Fraunhofer Institute for Casting, Composite and Processing Technology, Am Technologiezentrum 2, 86159 Augsburg, Germany; daniel.guenther@igcv.fraunhofer.de (D.G.); patricia.erhard@igcv.fraunhofer.de (P.E.); simon.schwab@igcv.fraunhofer.de (S.S.); 2 Faculty of Engineering, Ain Shams University, El-Sarayat Street 1, Cairo 11517, Egypt

**Keywords:** binder jetting, sands, vacuum thermoforming, fiber reinforced composite

## Abstract

Tooling, especially for prototyping or small series, may prove to be very costly. Further, prototyping of fiber reinforced thermoplastic shell structures may rely on time-consuming manual efforts. This perspective paper discusses the idea of fabricating tools at reduced time and cost compared to conventional machining-based methods. The targeted tools are manufactured out of sand using the Binder Jetting process. These molds should fulfill the demands regarding flexural and compressive behavior while allowing for vacuum thermoforming of fiber reinforced thermoplastic sheets. The paper discusses the requirements and the challenges and presents a perspective study addressing this innovative idea. The authors present the idea for discussion in the additive manufacturing and FRP producing communities.

## 1. Introduction

A main industrial focus today lies in energy-efficient and resource-saving manufacturing. This is one way to meet relevant ecological and economic targets as well as to ensure the competitiveness of products in the long term. As a result, lightweight components of high mechanical performance are increasingly attracting attention. Thus, fiber reinforced polymers (FRP), especially involving carbon fibers, are currently in the spotlight of developments. Related manufacturing processes, equipment and tools must be adapted to the new material and functionality requirements. A significant amount of time in the optimization process of a component lies in the creation of initial prototypes. The production of small series often proves to be particularly cost-intensive. These are primarily manufactured in manual processes. Depending on part complexity, costly and time-consuming tool manufacturing might be necessary.

Additive manufacturing provides a good solution regarding the rapid fabrication of tools. Next to functional integration, additively manufactured molds can combine complexity and lightweight. As an example, additive manufacturing makes it possible to realize complex cooling channels within the tool without the need of parting the mold. Some additive manufacturing techniques, such as the Binder Jetting process have a high volume throughput compared to the established CNC machining, thus allowing for lower manufacturing time and energy consumption. Further, additive manufacturing contributes to the conservation of material resources, since it relies on generating the final structure layer by layer as opposed to the material removal concept in established machining processes. For processing FRPs, both tools for low temperature applications (such as hand layup or Resin Transfer Molding) as well as high temperature applications (such as autoclave curing or compression molding) are relevant. Generally, tools do not require high strength [1,2]. In high pressure processes, as in the case of autoclave curing, compression molding or vacuum-assisted processes, loads are primarily compressive. In fact, these loads are often more favorable for additively manufactured parts, as compared to tensile loads [2].

Several studies have been concerned with the fabrication of tools by additive manufacturing from different materials. Brotan et al. [3] fabricated innovative tools out of Marlok C1650 steel powder in a Powder Bed Fusion (PBF) process to realize complex gradient structures to increase the thermal fatigue resistance and allow for a defined thermal management of the tools. The target applications of these low weight tools are injection molding and die casting. Similarly, Fette et al. [4] compared common additive manufacturing techniques—Additive Layer Manufacturing (ALM), Electron Beam Melting (EBM) and Selective Laser Melting (SLM)—with conventional methods to manufacture metal tooling. The authors claim that the use of integrated heating channels close to the mold surface allows the even dissipation of heat.

Warden [5] studied the application of Fused Deposition Modeling (FDM) to manufacture tools for compression molding thermoplastic multiaxial prepreg systems. The author proposed a low-temperature curing cycle in order to reduce the thermal degradation of the FDM tooling. Further, Bere et al. [6] processed Carbon Fiber Reinforced Polymers (CFRP) through vacuum bagging and oven curing using an FDM polylactic acid (PLA) versus acrylonitrile butadiene styrene (ABS) mold. The PLA mold was treated with a layer of epoxy that contains aluminum powder to enhance the bonding with a subsequently applied polyester gel coat, which further acts as a release agent and facilitates demolding. Hassen et al. [7] proposed the use of the extrusion-based Big Area Additive Manufacturing (BAAM) process to fabricate CFRP tools for further molding purposes. The main advantage lies in the increased throughput (~16,400 cm^3^/h) and the possibility to flexibly process material from granule form [8,9].

Further attempts have been made to reduce tooling costs for the thermoforming of non-reinforced plastics by additive manufacturing. Laser-sintered metal parts can be excluded due to their high costs. Although manufacturing costs can be reduced to as low as 14% compared to conventional tooling methods (milling), although this is offset by the high metal powder costs of 167% [10]. For the medical field, additive manufacturing of molds from calcium sulfate and gypsum powders was successfully demonstrated and even implemented for thermoforming of plastic structures [11,12]. In addition, Junk et al. [10] reported the application of the inkjet technology for thermoforming mold fabrication out of polymer gypsum. This was tested for a case study, in which a fairing made of ABS for an unmanned aerial vehicle was produced. Among other things, this study aimed to integrate vacuum channels in the manufacturing step without the need for additional post-processing (e.g., drilling). The study also explained the specific technical challenges, such as the demolding of undercuts or the separation of the tool. Results showed that the tool had sufficient strength for the subsequent thermoforming process. Another advantage of additive manufacturing is the design flexibility of both the overall geometry and the internal structure to influence strength and other physical properties for optimized vacuum guidance [11,13].

Little work has been carried out so far to elicit the advantages of using silica sand for tooling purposes. In this perspective, the authors claim that sand structures may prove to be efficient for tooling in general and for vacuum thermoforming processes in specific. Sand structures are thought to be cost-effective. Rough calculations evidenced a tooling cost of EUR 600 to 1000 for an aluminum or steel omega shaped tool compared to less than EUR 100 for a sand tool; the outer tool dimensions are 300 mm × 260 mm × 60 mm. In addition, their low thermal conductivity is favorable for the thermoforming process. First, maintaining the tool at constant temperature allows uniform manufacturing conditions for all parts that are to be formed. Second, when forming thermoplastics, the sheets need to be heated above their glass transition (for amorphous polymers) and slightly below their melting point (for semi-crystalline polymers). Within the process, it is necessary to ensure that forming is completed before the sheet temperature falls below these levels. Achieving that through low heat conduction through the tool may assist in allowing an energy-efficient thermoforming process. In the case of thermoforming, vacuum channels may be directly printed, eliminating the necessity of subsequent drilling steps. In addition, bonding of individual particles allows a porous structure, which might be advantageous for vacuum drawing.

This manuscript presents a novel idea and gives an insight regarding current and planned research activities targeting the use of sand molds for vacuum thermoforming of FRPs. The targeted manufacturing process for the mold is Binder Jetting. Further, the approach for mold modification for the anticipated application is presented. In addition, the challenges regarding the vacuum thermoforming of FRPs are discussed and a systematic procedure is followed to address them.

## 2. Thermoforming of Fiber Reinforced Composites

**Figure 1:**
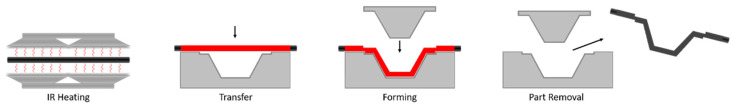
Schematic illustration of the thermoforming process.

One of the established methods in the automotive sector to fabricate FRP thin-walled structures is the Resin Transfer Molding (RTM) process. This involves the layup of multi-axial textiles into stacks, followed by draping into a 3-dimensional shape by matched tool pressing and finally resin infusion. This process is mostly limited to thermosets due to their low viscosity (below 1000 mPa∙s) compared to thermoplastics (beyond 100,000 mPa∙s) and is able to fulfill the demands for good surface quality. A drawback lies in the long cycle times needed for part curing (standard RTM processes may require several hours or days for curing; enhanced RTM processes 2–10 min) [14], thus limiting the use of thermosets for mass production. In contrast, the use of thermoplastic matrices makes short cycle times (60–140 s) [15] more feasible. Thermoplastics further have the advantages of providing improved toughness behavior and allow for recyclability. For fiber reinforced thermoplastics the thermoforming process is applicable. The process, as illustrated in Figure 1, implies forming a flat laminate into a 3D geometry under the influence of temperature. For heating IR lamps or convection ovens are often used [16]. The transfer time to the press has to be kept short in order to avoid extensive laminate cooling, which would prohibit adequate forming. For 3D forming of FRPs, both matched-die forming as well as deformable die forming is applicable. During shaping, the laminate is held in place using a support frame or a blank holder. This is crucial in order to keep the laminate under tension and accordingly prevent the creation of wrinkles. Further, the mold is kept closed during deformation to avoid heat loss and to maintain high pressures (10–40 bar), targeting proper compaction and full consolidation [17]. The final part is then left to cool within the mold, until it reaches a temperature below the glass transition temperature of the matrix.

Schug [17] summarized the main influencing parameters on the forming behavior and part quality. The author claims that the tool temperature has a major impact on the cooling time and morphology of the material. Further, the forming speed should be selected in such a way that allows a compromise between timely shaping and shear thickening response of the thermoplastic melt. The press load, where a high pressure favors consolidation on the one hand but may lead to dry spots and matrix flow on the other hand, mainly governs the surface quality. Further typical defects are fiber undulations, gap formation, out-of-plane wrinkles and folds [18,19]. Insufficient contact between mold surface and laminate at the end of the forming process may be the reason for rough surface areas. In the case of extensive tension of the laminate within the support frame, a fiber breakage and thus drastic losses in mechanical performance can be expected.

In the case of single and double diaphragm forming, pneumatic pressure is applied. The function of the diaphragm is to transfer the mechanical loads to the laminate and keeping a biaxial tension for wrinkle and undulation prevention. In single diaphragm forming, the laminate is placed over the mold having the diaphragm on top. The vacuum is applied and the laminate is forced against the tooling to take its shape. In double diaphragm forming the laminate is sandwiched between two flexible membranes and compacted by vacuum. Further vacuum is applied to press the sandwiched stack against the mold. This technique allows a greater degree of double curvature than single diaphragm forming [20]. For more intricate geometries and smaller fillets the hydroforming can be adopted. Here, a fluid pressure is applied to a rubber diaphragm to force the laminate stack to the mold. Hydroforming and diaphragm forming eliminate the need of mutual conforming tools and hence, the tooling cost is lowered [20].

Harrison et al. [21] investigated the use of springs instead of friction-based blank-holders to induce in-plane tension in the laminate in order to prevent the formation of wrinkles. This technique was evaluated as being flexible, easy to use and naturally facilitating heat transfer into the laminate prior to forming. However, the authors report that the focused application of tension might lead to in-plane buckling and localized zones of high shear.

**Figure 2:**
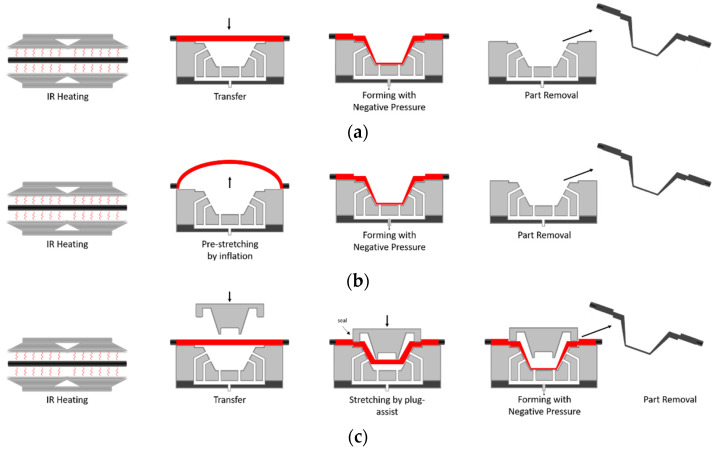
(**a**) Basic vacuum thermoforming process, (**b**) vacuum thermoforming with pre-stretching via inflation of a bubble, (**c**) vacuum thermoforming using a plug-assist.

The use of vacuum thermoforming for pure plastic sheets is a widespread process. In 2018, the market size for vacuum forming was estimated at USD 11.69 billion [22]. Various plastics (e.g., PA, PE, PP, PC or PET) are often applied. The basic process (Figure 2a) implies forming a sheet into a 3D geometry by heating it to a formable state, pressing it against a mold and holding it in that position until the sheet is cooled below the glass transition temperature. Finally, the part is trimmed to final shape [23]. In most cases, the processing temperatures are below the melting temperature and above the glass transition temperature (e.g., around 240–250 °C for PA6) [24,25]. Heating is mostly carried out by radiation, for example by using IR-heaters. Accordingly, bringing the plastic sheet to a viscoelastic state depends on the polymer’s ability to absorb the long infrared wavelength energy. Plastics with low crystallinity are said to have better formability [25] since the crystals hinder the flow behavior of the viscous fluid.

Despite its high process flexibility and good suitability for prototype and series production, thermoforming is associated with some challenges. When using single-sided tools, the component thickness can vary significantly, due to varying stretching according to the geometry. Sheet areas first touching the mold are hindered in their biaxial motion by friction and thus remain thicker than other areas that continue to witness stretching. This is also associated with faster cooling by heat conduction from the touching area to the mold. This uneven deformation and cooling behavior can lead to residual stresses in the final component [23]. As a remedy, a pre-stretching step may be introduced after the heating step. In that case a clamping tool fixes the edges of the sheet and air pressure is applied to inflate the sheet into a bubble (Figure 2b) [26]. In the next step, forming is achieved by vacuum pressure. Nevertheless, the thickness distribution in the final components largely depends on the viscoelastic properties of the polymer materials.

In plug-assisted (Figure 2c) thermoforming, the heated sheet is pre-stretched through a mechanical plug. Next, actual forming is completed through the application of vacuum pressure [27]. Chen et al. [28] observed that thickness at the sidewalls increased with increasing mold temperature, preheating temperature, plug depth and holding time, but decreased with increasing plug speed. At a thermoforming temperature above the glass transition the sheet material becomes sticky and thus unable to slip over the plug surface due to increased friction [27,29,30].

## 3. Binder Jetting of Sand Tools

Conventional tool manufacturing relies on subtractive (machining) process starting from a material block. Such tools are often metallic (e.g., tool steel type AISI-SAE 1045 [31]) or in some cases made from polymers (e.g., RAKU^®^Tool [32]) (RAMPF Tooling Solutions GmbH & Co. KG, Grafenberg, Germany) and foams. Considering material costs and machining time, the fabrication of tools may prove to be extensively time consuming and costly [31].

**Figure 3:**
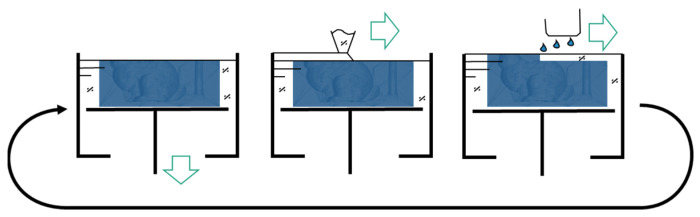
Schematic illustration of the steps characterizing the Binder Jetting process: First—lower platform, Second—recoat layer, Third—jet binder.

In contrast to the subtractive manufacturing, additive manufacturing builds the structure in a layer-by-layer technique, applying material only there where it is needed. Figure 3 illustrates the Binder Jetting process according to ASTM [33]. Here, the following process steps are repeated until the desired component is created: A build platform is lowered by a layer thickness of ranging between 10 µm to 400 µm. The hereby-created free space is then filled with powdered material using a re-coater. In the third step, a binder is selectively deposited using an inkjet print head to bond the individual particles together. This creates a bond within the layer and with the layer below. In the case of Binder Jetting of sand, sand grains are used as the building material.

This setup makes Binder Jetting systems particularly easy to scale in terms of performance. The number of nozzles correlates with the overall performance [34]. Likewise, several layers can be applied simultaneously. This feature cannot be achieved with laser-based systems using beam deflection. Such unique arrangement leads to extraordinary build rates and, consequently, to special cost-effectiveness. Binder Jetting currently achieves a maximum build-up rate of approximately 400 L/h (Datasheet Exerial^TM^ 3D) (ExOne, Gersthofen, Germany) printer and costs significantly less than 10 €/l. The basis of the Binder Jetting can be various particle materials. Plastic particles, metal powders or inorganic materials such as sand or ceramics can be used. The particles are adapted to the layer thickness to be achieved, and the spectrum of average particle sizes ranges from d_50_ = 20 µm to d_50_ = 400 µm. Silica sand is particularly cost-effective for use as tools. Here, the raw material costs are often less than 100 €/t.

The components manufactured by Binder Jetting can be used either directly or with post-treatment as a tool. Post-treatment usually increases the strength and wear resistance of the product. Used directly, sand binder systems achieve a maximum tensile strength of about 10 MPa (identified by preliminary examinations and further referred to as the base material). The strength can be significantly increased, for example, by infiltration with an epoxy resin. The sand or the underlying particle material can be sintered or an impression can be made with a higher-strength material (e.g., with a polymer cement) when even higher strengths are required.

**Figure 4:**
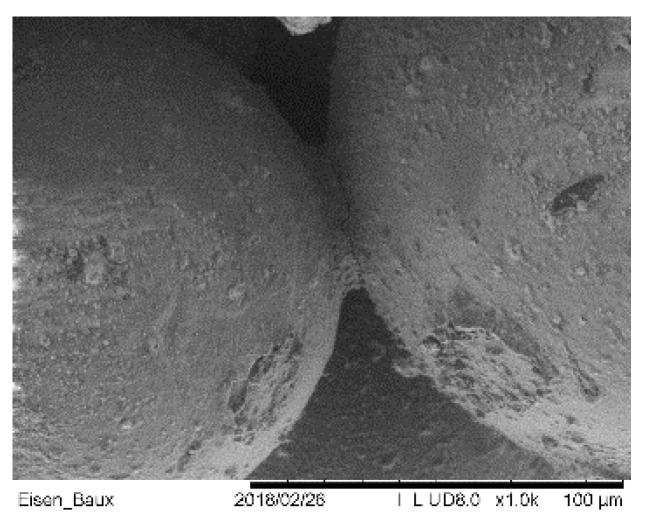
SEM-Micrograph of the connection between two grains.

The strength of the base material is affected by different aspects related to material and process, such as the strength of a sand grain, the density of the sand, the binder adhesion and cohesion as well as the amount of binder. The sand grains are packed more or less densely during the coating process, depending on the technique [35]. This results in a volume ratio of air to sand of about 50%. Depending on the binder system, approximately 2–5 wt% binder penetrates into the loose sand during the deposition of the binder. The capillary action causes liquid to accumulate at the contact points of the sand grains (Figure 4), which solidify by drying or polymerization, depending on the binder system. The cavity that now remains can be filled by infiltration with another material system, thus raising the strength due to the higher amount of binder compared to the base material.

The properties of printed sand molds can be used especially in applications where low strength is of secondary importance or even helpful. For example, a casting core needs to be as strong as necessary for safe handling but, after the casting process, has to be easily removed out of the cavity. Also relevant here are cores for hollow structures that have to be removed after the lamination process (washouts) [36].

**Figure 5:**
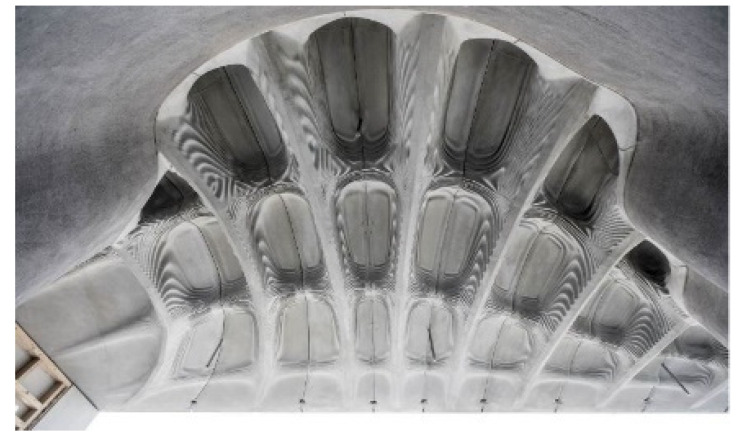
Concrete part formed on a Binder-Jetted tool [38].

Tools for casting purposes are also manufactured using tools fabricated by Binder Jetting. Very different casting materials can be used in this process. For example, concrete can be molded to produce structures in the construction industry (Figure 5) [37,38]. The molds must be pre-treated for casting by sealing the surface to prevent the ingress of mixing water. After the concrete has set, the mold is opened and removed from the structural member. Depending on whether the structure is undercut, the molds can be used once or several times. This is also the case, with laminating molds where the mold is used only for a few impressions in prototyping applications.

Sand molds are further widely spread in the foundry industry for the production of metal castings. Here, individual parts with small and large dimensions are often realized with printed molds. In particular, parts that conventionally require the storage of large molds over long periods can be produced economically using Binder Jetting [39]. On the other hand, large series in engine construction can nowadays also be realized with sand molds from 3D printers [40].

**Figure 6:**
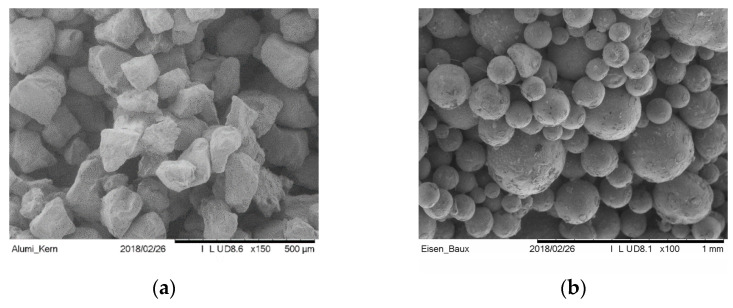
SEM-Micrographs of different qualities of sand suitable for the Binder Jetting of tools: (**a**) Silica Sand type GS14 RP by Strobel Quarzsand GmbH Freihung, Germany, and (**b**) Bauxitsand by Hüttenes Albertus GmbH Düsseldorf, Germany.

Various sand types have been qualified for the usage in Binder Jetting of sand molds and cores [41]. Silica sand is most commonly used in foundries to make molds (amongst other methods also through additive manufacturing), due to its cost effectiveness. The sand morphology and particle size (Figure 6) distribution are known to strongly affect the resulting packing density [35] and surface properties [42]. The packing density influences both mechanical strength and permeability. In general, by choosing a sand with a smaller d_50_ medium grain size than a reference one, a higher casting surface quality can be expected [43]. However, this grain size implies low permeability to air and gases. The shape of the sand grain also has a decisive influence. Sharp edged grains, on the one hand, have the least contact with each other in a compacted structure and thus make the sand highly permeable to gases. On the other hand, they cannot be packed to the optimum extent during Binder Jetting and structures made from them have low strength [44].

Current binder systems in combination with sand as a molding material are inexpensive epoxy, furan or phenolic resin–based systems that bring sufficient thermal stability. Environmentally friendly inorganic binders are nowadays the subject of research and are preferred within this study [43,44].

For 3D printing of tools, the mechanical properties of the 3D-printed part prior to infiltration might be of minor importance. Thus, a sufficient strength for secure handling during the infiltration process is expected to be acceptable considering the overall process. The permeability, however, is essential to ensure a sufficient infiltration depth and thus to provide the basis for infiltrated sand tools of enhanced mechanical properties. Sand qualities that will be investigated within this study are silica sands obtained from natural reservoirs of varying powder size distributions as well as artificial sands produced by sintering. In contrast to natural sands, artificially produced sands typically show regular, round shapes and narrow particle size distributions.

## 4. Sand Tools for Vacuum Forming of FRP

The main target of the investigations is to develop an innovative process chain for the rapid and cost-effective production of large-area thermoplastic-based shell structures made of fiber reinforced plastics—especially during the product development phase, or where small part numbers are needed. In the solution approach, the advantages of additively manufactured sand molds are combined with the possibilities of a fast thermoforming process. This results in synergy effects that significantly advance prototype and small series production. In the following the main requirements regarding sand tools for vacuum thermoforming applications are given:

- Compression strength exceeding 20 MPa in order to stand the specific process loads;
- Acceptable wear resistance to withstand at least ten forming cycles;
- Surface finish with arithmetic mean roughness R_a_ < 20 µm to minimize indentations on visible surfaces;
- Porous structure to ensure a safe application of vacuum;
- Temperature resistance up to 280 °C;
- Cost-effective in terms of engineering and production comparable with state-of-the-art tools.

**Figure 7:**
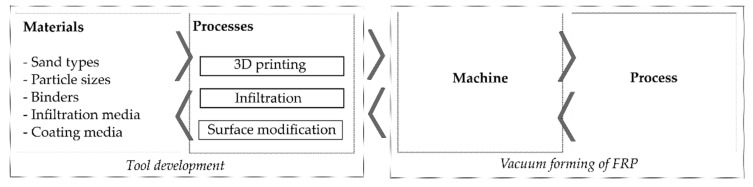
Investigation approach targeting the fabrication of sand tools for vacuum forming of FRP.

In order to address the aforementioned requirements, a segmented approach is applied (Figure 7). In the first stage, the focus is laid on the sand mold, where materials and process parameters will be defined. After 3D printing by Binder Jetting, a mockup tool is further treated to fulfill the requirements regarding mechanical properties and surface quality. In parallel, the feasibility of vacuum thermoforming is studied, addressing the main challenges such as the applicable clamping mechanism for FRP laminate to ensure vacuum pressure on the one hand and draping of the laminate under tension on the other hand.

### 4.1. Initial Approaches and Results in Binder Jetting of Sand Tools

**Figure 8:**
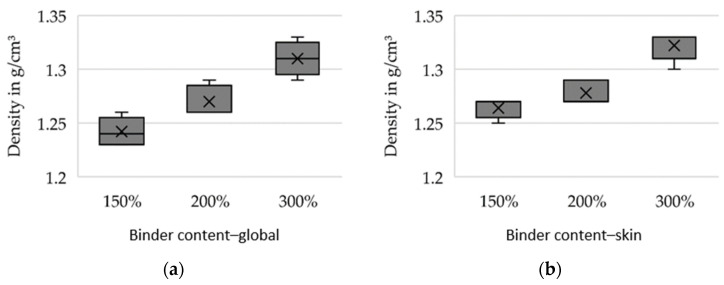
Density of specimens produced at varying binder contents. The binder content was increased (**a**) globally and (**b**) only in an outer skin of 5 mm.

**Figure 9:**
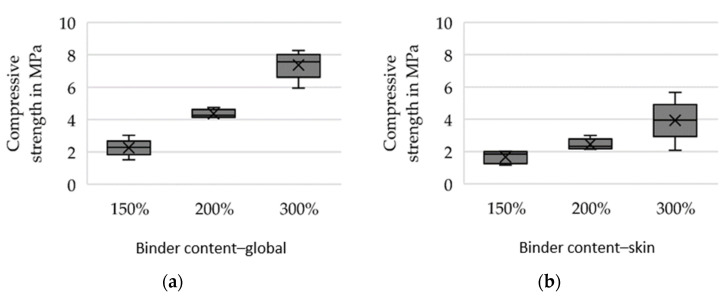
Compressive strength of specimens produced at varying binder contents. The binder content was increased (**a**) globally and (**b**) only in an outer skin of 5 mm.

**Figure 10:**
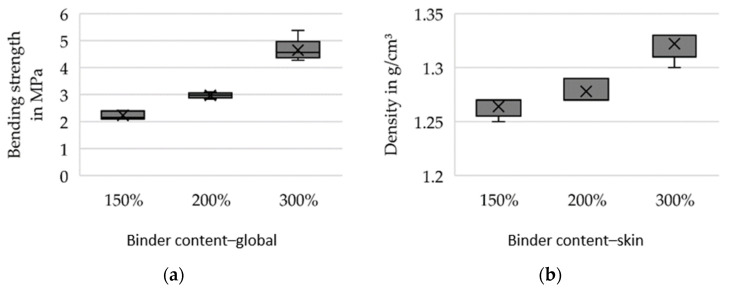
3-point bending strength of specimens produced at varying binder contents. The binder content was increased (**a**) globally and (**b**) only in an outer skin of 5 mm.

For preliminary tests, the silica sand GS14 (Strobel Quarzsand GmbH, Freihung, Germany) in combination with an inorganic binder system (IOB by voxeljet AG, Friedberg, Germany) was selected. The 3D printing tests were carried out using increased binder weight contents (150%, 200% and 300% with respect to conventionally used binder amount) in order to investigate the feasible limits and their effects on the mechanical properties considered relevant for sand tool fabrication for vacuum forming of FRP. Further, during 3D printing, the binder content is to be increased globally on the one hand and selectively varied locally on the one hand. Local variation (further referred to as the skin-core setup) implies the fabrication of a skin layer of 5 mm thickness with high binder content relative to the core segment. This allows for superior mechanical properties at the mold surface. In contrast, in the case of global binder application at high binder content, the entire sand structure is printed at the aforementioned increased values. Two sets of specimens were produced: bars with a cross-section of 22.4 × 22.4 mm for 3-point flexure tests and cylinders D50 × 50 mm for compression tests and density evaluation. Five specimens were tested for every parameter set. Figure 8, Figure 9 and Figure 10 show the results of the preliminary investigations, indicating that the increase of binder content can be directly correlated with an increase in density, compressive strength and bending strength for globally increased binder contents (a) and skin–core settings (b). The specimens’ densities printed using the skin-core strategy were found to be higher than those of the respective specimens with overall increased binder contents (Figure 8). This effect was attributed to the fact that the printed binder of the underlying layer and its residual moisture impedes the deposition of new powder and its compressibility. The compressive strengths of the specimens was significantly reduced by the skin–core setting (Figure 9), while the bending strength slightly increased (Figure 10). This was related to the maximum bending stress, which increases indirectly proportional to the geometrical moment of inertia of the cross-section that is smaller for hollow rectangular cross-sections compared to filled rectangular areas.

Especially the dimensional accuracy in building direction decreased significantly with increasing binder contents. While the specimens’ dimensions (measured by a Vernier caliper) in building direction roughly comply with targeted dimensions for moderately increased binder contents (mean deviations of +0.83% for 150% binder content and +1.56% for 200% binder content), the dimensions for 300% binder content deviated extremely from the targeted values (mean deviation of +3.91). The upper application limit of binder content was therefore identified to be 200% for the observed sand–binder combination. Especially the print strategy (e.g., the local density of the jetted binder), were found to play an important role and will be further investigated.

### 4.2. Initial Approaches and Results in Infiltration of Sand Tools

**Figure 11:**
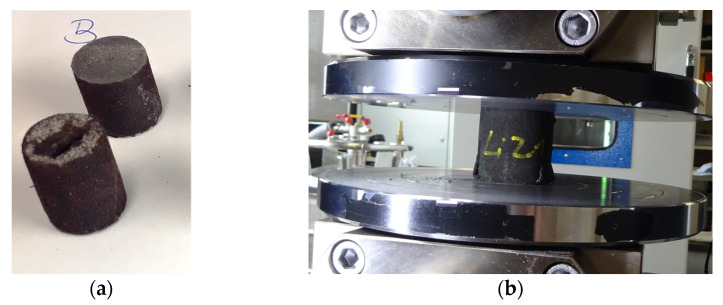
The 3D-printed specimens infiltrated with epoxy resin: (**a**) fully infiltrated specimens of dimensions D15 × 20 mm, (**b**) spalling effect during compression testing for specimens of dimensions D50 × 50 mm.

The requirements regarding the mechanical properties cannot be achieved with the basic material system. A conflict of targets exists regarding strength, penetrability and final permeability. This conflict can only be solved by considering the process, i.e., including printing and infiltration. Therefore, the 3D-printed sand structures were subsequently impregnated with various resins. In a preliminary test, first promising results were gained by infiltrating two D15 × 20 mm specimens. The samples were Binder-Jetted using GS14 sand and furan resin (VX-2C by voxeljet AG, Friedberg, Germany). The Epoxy resin (IH16 by Ebalta Kunststoff GmbH, Rothenburg ob der Tauber, Germany) was applied as the infiltration medium. Infiltration was carried out manually at room temperature using a brush. Figure 11a shows two infiltrated specimens: at the top of the figure, a radial cut shows the full impregnation of the sand specimen; at the bottom, the second specimen, previously tested for compression strength, is shown. The fully infiltrated specimen reached a compressive strength of ~70 MPa, thus exceeding the targeted value of 20 MPa. However, when altering the specimen dimensions to D50 × 50 mm, it was observed that only a comparably limited penetration depth could be achieved. Figure 11b demonstrates that such samples witnessed a spalling effect during compression testing. This was accompanied by a reduced mean compression strength of 5.9 MPa at a standard deviation of 0.3 MPa. When doubling the amount of infiltration medium for another five specimens of D50 × 50 mm, spalling could still be observed, while the compression strength increased to a mean compression strength of 12.1 MPa at a standard deviation of 3.1 MPa. These first experiments were intended to show the potential and challenges associated with the infiltration of 3D-printed sand structures. Further examinations with a larger quantity of specimens including a broader variety of materials are planned for future investigations.

A rough evaluation of the surface quality achievable by grinding the epoxy-infiltrated structure showed R_a_ of 7 µm, fulfilling the requirements stated above. However, hand-grinding using sand 120-grit paper shows a low level of automation. Thus, alternative methods are also scope of the future investigations.

### 4.3. Intended Future Investigations Regarding Sand Tool Fabrication

The results of the first investigations showed that the requirements regarding the tools can in principle be met. The preliminary tests have to be refined in order to prove the feasibility of the production of suitable tools.

The planned study implies the evaluation of the mechanical performance, dimensional accuracy, density, permeability and surface quality of the sand structures with respect to the targeted requirements. Table 1 presents the applied test methods for evaluation.

**Figure 12:**
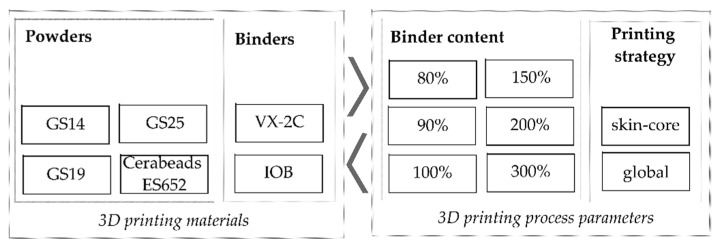
Planned variations regarding 3D printing of sand tools.

Various sands are to be considered for the future study in order to investigate the effect of particle size and shape on density, strength, roughness, penetrability and permeability. Three different particle size distributions of natural silica sand will be investigated (GS14, GS19 and GS25 by Strobel Quarzsand GmbH, Freihung, Germany 3D) as well as one synthetic sand composed of aluminium silicate (Cerabeads ES650 by Hüttenes-Albertus Chemische Werke GmbH, Düsseldorf, Germany). Printing will be carried out using two different binder systems: a furan resin (VX-2C by voxeljet AG, Friedberg, Germany) that is the most frequently used for Binder Jetting of sand molds and an inorganic binder system (IOB by voxeljet AG, Friedberg, Germany), which is gaining increased attention due to its low emissions during casting. Further, a graded structure that allows for a controlled permeability for subsequent impregnation and the final vacuum forming purposes may be applicable in sand tools. Therefore, the binder content and the printing strategy will be further investigated in accordance with the preliminary tests. In addition, the lower limit regarding binder content will be identified. This is expected to result in lower densities, which may enhance the penetrability of the 3D-printed sand structure by the infiltration media. Figure 12 sums up the planned investigations with the various material systems and 3D printing parameters.

**Figure 13:**
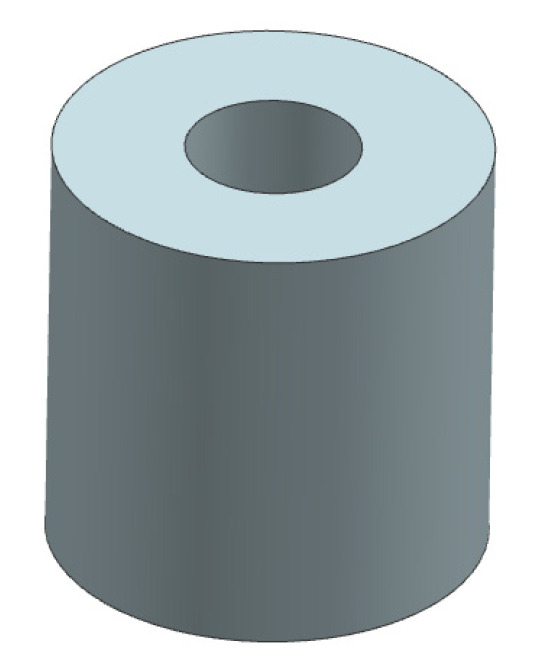
Suggested specimen geometry for the determination of the penetration depth.

Further investigations target the enhancement of the penetration depth using alternative resins of higher penetrating capacity in addition to tailoring the permeability of the 3D-printed structure by locally altering the binder content. Figure 13 shows the suggested specimen geometry designed for investigating the depth of penetration. The sample is cylindrical with a central hole making an indentation of 5 ml volume. Accordingly, a pre-metered amount of infiltration media is to be applied into the indentation, which will allow a comparability of the impregnation efficiency with varying infiltration media at varying temperatures and printing material systems (involving powder and binder) and parameters. The penetration depth will be examined microscopically across the cross-section.

Further investigations will include surface modifications, as smooth surfaces will not only enhance the surface quality of the FRP part but also are expected to reduce forces when demolding. Investigations will concern known application methods like grinding and coating of the infiltrated tools as well as the modification of the impregnation media.

### 4.4. Thermoforming of Organosheets

The implementation of vacuum thermoforming for FRPs requires a step-by-step approach to overcome the first obvious challenges. These mainly lie in the lack of ductility of the fibers in contrast to the surrounding thermoplastic matrix. Heating the organosheet above the melting point of the matrix allows the matrix to flow and thus to be formed. During this stage, the matrix is stretched within the blank holder or the support frame. In the case of endless fibers, these are tensed but remain unable to stretch. This leads to the withdrawal of the organosheet from the support frame or excessive folds. Accordingly, a flexible holder is necessary; this should allow the sheet to flow and feed new material necessary to envelope the 3D mold geometry.

**Figure 14:**
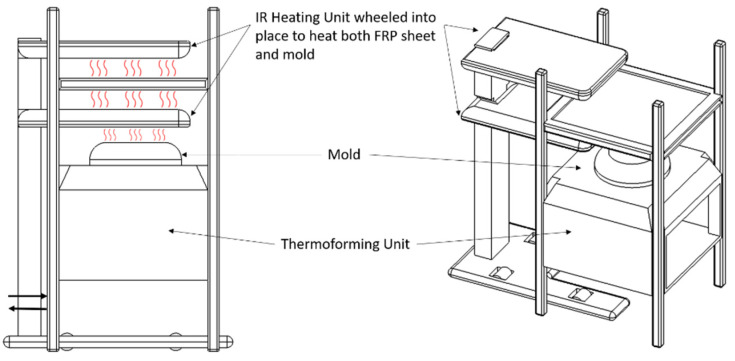
Preliminary concept of the heating and thermoforming unit.

A further challenge lies in the load available for deformation. It can be expected that the vacuum pressure alone is insufficient for deforming FRP sheets. Although the main target of the study is to keep the process as simple as possible, it is kept in mind that an application of a diaphragm might prove to be inevitable. Other than in the case of conventional thermoforming with unreinforced polymer sheets, it is assumed that heat must be applied to both surfaces of the sheet in order to achieve a homogeneous melt of the matrix throughout the thickness. This implies that the setup of a thermoforming unit must be adapted to allow heating both sheet surfaces in addition to the mold surface in order to avoid rapid cooling of the sheet during deformation. Figure 14 shows the envisaged machine setup. An IR-heating unit is moved into the thermoforming unit, to simultaneously heat both sides of the sheet and the mold surface. The heating unit is then wheeled out and the thermoforming process takes place.

**Figure 15:**
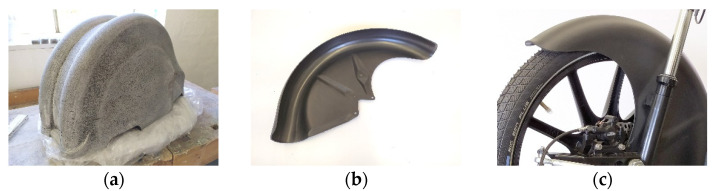
(**a**) Binder-jetted sand mold for thermoforming thermoplastic fenders, (**b**) thermoformed fender made from ABS, (**c**) application of the ABS fender.

In order to tackle these challenges, first, the focus was set on the development of the sand mold for vacuum thermoforming applications in general. Hence, the first tools developed in the above-mentioned stage were tested for their feasibility for pure thermoplastic sheets. In that stage, an ABS fender was vacuum thermoformed. The sand mold, illustrated in Figure 15a, is binder-jetted as a hollow structure in order to save weight (final weight 30 kg) and equipped with vacuum channels. The mold was further impregnated using an epoxy resin to increase the stability of the surface layer. For improved surface finish, the mold was ground using sandpaper (grit size 120). The process proved to be feasible and the surface quality, as presented in Figure 15b,c, was found acceptable.

**Figure 16:**
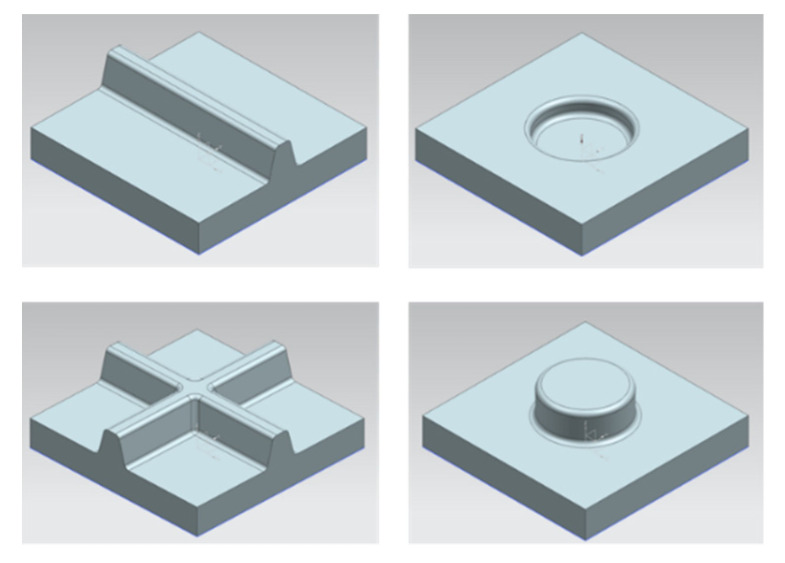
Targeted designs of mockup molds to tackle the challenges in various complexity levels.

In the next phase, the processing ability of FRPs is studied using metallic molds equipped with conventional vacuum channels. Here, issues like vacuum pressure, heating temperature, heating time and setup are examined. The difficulty level is increased by first considering randomly oriented short fiber reinforced sheets. Then, the fiber length and the orientation are altered step by step. When considering endless fibers, the behavior of woven textiles will be observed in contrast to multi-axial fabrics. These experiments are to be carried out on different levels of geometrical complexities. Figure 16 shows the envisaged geometries of the mold.

As mentioned above, the support frame has to fulfill several functions and resembles one of the key factors for successful thermoforming. First, the frame has to seal the forming area to ensure vacuum build-up. Further, it should act as a blank holder that has to provide enough degrees of freedom to allow for feeding sufficient sheet material to compensate the desired 3D shape. Only if both features are provided by the clamping mechanism a good molding process and part quality can be expected.

**Figure 17:**
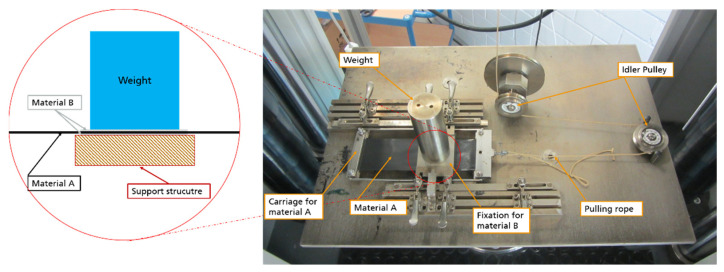
Test rig for measurement of frictional forces between FRP sheet and clamp materials.

In order to be able to identify the necessary clamping force, preliminary evaluation of the frictional forces between the above-mentioned materials and the anticipated frame materials were conducted. The investigations were performed using a self-constructed test rig, as depicted in Figure 17. The rig consists of a carriage for material A and a further fixture for material B. Material A is cut to the dimensions 180 mm × 50 mm and is clamped at both ends into the carriage. Material B is prepared to the dimensions 110 mm × 50 mm. The test setup involves sandwiching two layers of material A between two single layers of material B on each side (Figure 17 magnification). Hence, a contact area of 50 mm × 50 mm is created. The normal (press) force is controlled by weights. For the measurements, the test rig is mounted into a universal testing machine. The pulling rope is attached to the crosshead and translates the vertical movement into a horizontal movement between material A and B through the idler pulleys. When the carriage is moved back and forth, a friction between A and B can be measured.

The candidate materials for the support frame in direct contact with the sheets were selected to be polytetrafluoroethylene (PTFE) and aluminum. Regarding the anticipated sheet materials, a unidirectional carbon fiber and another short-recycled carbon fiber reinforced polyamide tape were investigated. The material combinations examined are summarized in Table 2. For this test campaign, the universal test machine ZwickRoell Z100 in combination with a KMD 5 KN load cell was used. For creating a specific press force between materials A and B, a 1 kg mass was applied on the materials. The crosshead was moved with a constant speed of 100 mm/min and stopped after a displacement of 80 mm. All tests were performed at room temperature. The load-displacement data were recorded for three replicates, where only material A was replaced by a new one after each examination. To calculate the friction coefficients, two forces were evaluated from the load-displacement curves. The static and dynamic coefficients of friction were calculated according to Equation (1), where µ_PT_ is the coefficient of friction, F is the recorded force, F_N_ is the applied normal force, m is the mass and g the gravitational acceleration.

In the case of the static friction coefficient µ_PTs_, the force F is identified at the beginning of motion as the maximum force value between 0 and 10 mm displacement, whereas for the dynamic friction coefficient µ_PTd_ F is given by the average force value, between 10 and 60 mm displacement. The calculated values are presented in Table 2.

(1) μPT=F2∗FN; FN=m∗g=1 kg∗9.81 m/s^2=9.81 N

**Figure 18:**
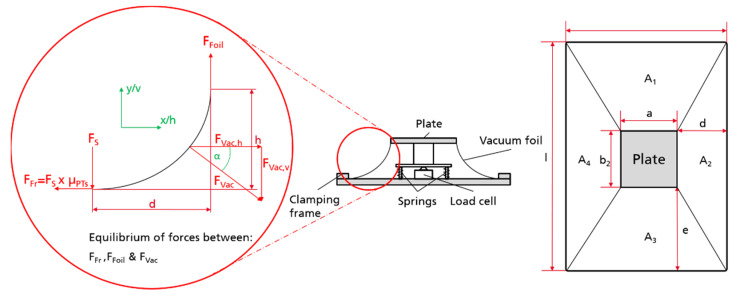
Side view of the test stand and acting forces and top view of the test stand.

A simple model for the calculation of the clamping force was developed based on the coefficients of friction µ_PT_, the achievable vacuum pressure and the required forming geometry. The model is presented in more detail in the Appendix A. Through the known pressure difference ∆p (between vacuum and ambient pressure) and the surface area A of the sheet, the resulting force F_vac_ can be calculated. With this force and the assumption that the tape behaves like a rope (inelastic material, unable to transfer moments), the clamping force F_s_ can be estimated. Accordingly, Figure 18 gives a simplified estimation of the forming process at its initial state, where the pliable sheet is not yet in contact with any of the mold surfaces except the top surface (indicated by the plate in Figure 18).

**Figure 19:**
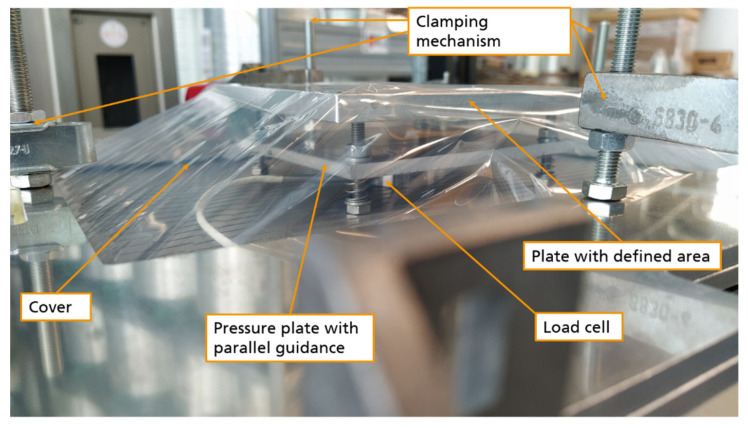
Test setup for the validation of the developed model.

The test rig is further illustrated in Figure 19. Knowing the inclination angles and dimensions of the test rig, the surface area of a clamped foil can be calculated. Further, the vacuum force F_vac_ applied to the foil can be determined and can be decomposed in its horizontal and vertical force components, for each area segments (A_1_–A_4_ in Figure 18). Based on the assumption that the tape behaves like a rope and that the tape does not transport any momentum in addition to the boundary condition, that F_s_ × µ_PTs_ < F_Foil_ (where F_Foil_ is the tangential tensile force in the foil), the parameters for all area segments can be calculated.

To validate the model and calculations, a test (Figure 19) was performed, using the aforementioned setup, while integrating a load cell in an exact copy of the described model (Figure 18). A PA6 foil (commonly used in vacuum infusion processes) was used. A vacuum cleaner (Wieland IS-46h) was used to create the desired pressure difference of 270 mbar. After assembling the test stand and clamping the foil in the frame the load cell is tared, and the vacuum was applied. A nominal force of 0.91 kN was generated and was further used to validate the model. The first trials indicated that with the vacuum cleaner and the test stand the expected pressure difference of 270 mbar could not be achieved. However, the pressure difference was not measured, and it remains unclear what the absolute achieved vacuum pressure would lead to, regarding the pressure difference between ambient and vacuum. This difference is vital for the calculations. Hence, in the next step, a pressure sensor is integrated into the test stand to raise the necessary data for validation.

Once the theoretical model is validated, it can be applied to construct the support frame and to define the necessary clamping forces. These aspects will further be considered for a prototype vacuum thermoforming stand. The products, produced with the prototype machine, are to be evaluated regarding their dimensional accuracy, the fiber distribution and local fiber volume content.

**Figure 20:**
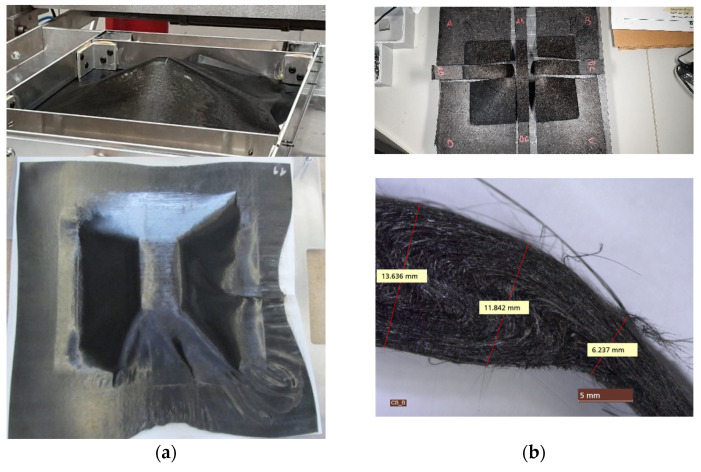
(**a**) Vacuum thermoforming of a multiaxial endless CFRP sheet under non-optimal conditions. (**b**) Vacuum thermoformed short CFRP sheet and resulting lofting effect.

First experimental trials have proved the above-mentioned challenges. Trials with endless fiber reinforced thermoplastic sheets (Figure 20a) confirmed the demand for a new clamping concept and a two-sided heating system with short transportation routes from the heating unit to the forming unit. Further, the trials have shown, that the short fiber material can be challenging, as it tends to loft (Figure 20b) and thus generate voids in the process, which in turn negatively affect the generation of the required vacuum pressure.

### 4.5. Summary of Technical Challenges

Preliminary investigations showed that 3D-printed sand specimens meet the requirements for the application of FRP vacuum forming tools regarding strength and surface quality on laboratory scale. It was shown that it is possible to customize density and strength by adapting the 3D printing parameters and through post-processing by infiltration, while varying impregnation parameters and media.

Technical challenges in developing 3D-printed sand tools for vacuum forming of FRP derive in particular from increased mechanical and thermal loads when forming FRP sheets. Process related mechanical peak loads are expected to occur during forming and ejecting of the formed part resulting in multi-axial stresses on the tool. Thus, a geometry-dependent stability of the tool material is needed. However, a residual permeability is expected to be advantageous for the vacuum-forming process. Especially the homogeneity of the material properties within the tool, strongly influenced by the infiltration process, are expected to play an important role for the long-term stability of the tools.

Moreover, temperature increases in the tool arise along with an increased number of production cycles as the sheets solidify in direct contact with the tool surface. However, using 3D printing offers extensive possibilities to overcome constraints by including enhanced functionalities, such as undercut cooling and vacuum channels.

Regarding the vacuum forming of FRPs, the main challenges lie in the construction of a suitable sheet holder that allows material feed to compensate its draping over the mold and that provides enough tension to prohibit wrinkles in the sheet. In addition, rapid cooling against the mold has to be prevented up to the point, where the geometry is accurately mapped. Further, the forming process itself, the dynamic process and the reproducibility of the material feeding behavior will be the key challenges for successful vacuum forming.

In view of the transfer to an industrial application, the central key challenge is defining an economically, sustainable and preferably automatable process for the production of tools of consistent quality.

## Figures and Tables

**Table 1:** Materials testing methods for further investigations.

Material Testing Method	Standard
3-point flexure test	VDG P71
Compression test	DIN EN ISO 126
Dimensional accuracy	DIN 862
Permeability test	VDG P41
Density determination	DIN 862, DIN 8128-1
Roughness measurement	ISO 4288

**Table 2:** Overview of the material combinations, the measured forces and calculated friction values.

Material Combination		Average Max. Force [N]	Average Sliding Force [N]	$\mu_{\mathrm{PTs}}$ (Static Friction)	$\mu_{\mathrm{PTd}}$ (Dynamic Friction)
Material A	Material B				
UD CF-PA	Teflon	4.78	3.06	0.243	0.156
UD CF-PA	Aluminium	6.91	5.08	0.352	0.259
UD CF-PA	UD CF-PA	4.68	3.7	0.238	0.189
Teflon	Aluminium	8.71	6.3	0.444	0.321
rCF_PA	Teflon	5.89	4.35	0.300	0.222
rCF_PA	Aluminium	12.37	7.02	0.624	0.358

**Figure A1:**
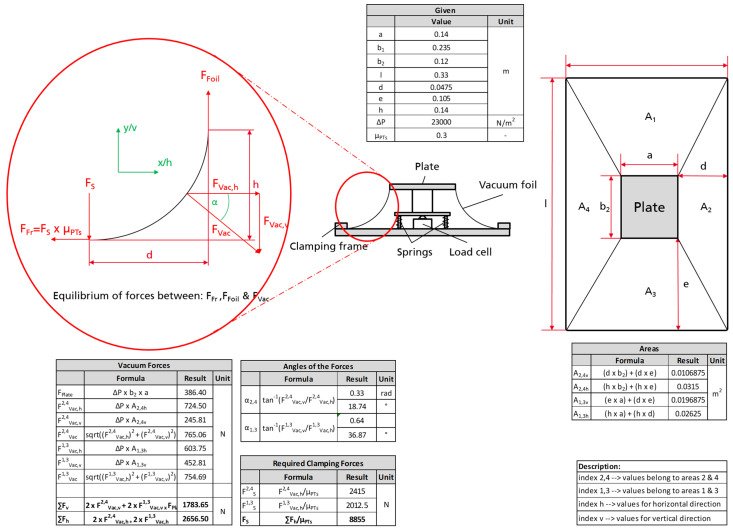
Model for the Calculation of the Necessary Clamping Force.

## Data Availability

Not applicable.
